# Prognostic role of effective radiation dose to immune cells in esophageal cancer treated with definitive chemoradiation

**DOI:** 10.1371/journal.pone.0336794

**Published:** 2025-11-13

**Authors:** Yoo Kyung Choi, Seok Hyun Son, Hong Seok Jang, In-Ho Kim, Sea-Won Lee, Soo-Yoon Sung

**Affiliations:** 1 Department of Radiation Oncology, Eunpyeong St. Mary’s Hospital, College of Medicine, The Catholic University of Korea, Seoul, Korea; 2 Department of Radiation Oncology, Seoul St. Mary’s Hospital, College of Medicine, The Catholic University of Korea, Seoul, Korea; 3 Division of Medical Oncology, Department of Internal Medicine, Seoul St. Mary’s Hospital, College of Medicine, The Catholic University of Korea, Seoul, Korea; All India Institute of Medical Sciences, INDIA

## Abstract

**Background:**

Radiotherapy for locally advanced esophageal cancer can induce lymphopenia, potentially worsening outcomes. This study examines the association between clinical outcomes and the effective dose to the immune cells (EDIC), a measure of lymphocyte radiation exposure.

**Methods:**

We retrospectively analyzed 107 patients with locally advanced esophageal squamous cell carcinoma treated with definitive concurrent chemoradiotherapy (CCRT). The EDIC was calculated based on the mean lung dose, mean heart dose, and integral total body dose using established models. Patients were stratified into high (n = 42) and low (n = 65) effective dose to the immune cells (EDIC) groups using a cut-off value of 4.28 Gy. Survival outcomes, including overall survival (OS), progression-free survival (PFS), locoregional failure-free survival (LRFS), and distant metastasis-free survival (DMFS), were assessed.

**Results:**

The 5-year OS and PFS rates were significantly lower in the high EDIC group than in the low EDIC group (51.9% vs. 66.6%, *p* = 0.043; 20.8% vs. 31.8%, *p* = 0.002, respectively). Multivariate analysis identified high EDIC as an independent predictor of poorer OS (hazard ratio (HR): 2.06, 95% confidence interval (CI): 1.1–3.86, *p* = 0.024) and PFS (HR: 1.7, 95% CI: 1.04–2.78, *p* = 0.034). Similarly, the 5-year LRFS and DMFS rates were significantly lower in the high EDIC group than in the low EDIC group (24.1% vs. 34.9%, *p* = 0.003; 29.0% vs. 44.0%, *p* = 0.018, respectively).

**Conclusion:**

A higher EDIC is an independent predictor of poor survival in patients with esophageal squamous cell carcinoma undergoing CCRT. Reducing radiation exposure to the immune system through optimized radiation planning and lymphocyte-sparing techniques may improve patient outcomes.

## Introduction

Esophageal cancer is one of the most lethal malignancies worldwide, ranking as the sixth leading cause of cancer-related deaths. It has a particularly high incidence in Eastern Asia, where squamous cell carcinoma is the predominant histological type [1–3]. Despite advancements in treatment, the overall prognosis remains poor, with a 5-year survival rate of approximately 20% across all stages [4,5].

Definitive concurrent chemoradiotherapy (CCRT) is the primary treatment option for locally advanced esophageal cancer, especially for patients ineligible for surgery [6–8]. Radiotherapy (RT) plays a critical role in the management of this disease; however, treatment fields often extend from the supraclavicular lymph nodes to the upper abdomen, covering a large area that includes normal organs such as the lungs and heart [2,9].

The effects of RT extend beyond solid organs and significantly affect the immune system. In particular, lymphocytes are highly sensitive to radiation, with substantial depletion occurring even at relatively low doses (<1 Gy) [10]. Radiation-induced lymphopenia can lead to impaired immune function and negatively influence the prognosis of various cancer types [11–16]. These changes can weaken immune surveillance of the body, thereby diminishing the antitumor response. Therefore, there is growing interest in understanding the systemic effects of RT on lymphocytes and prognosis.

In a secondary analysis of the RTOG 0617 trial, a metric known as the effective dose to the immune cells (EDIC) was introduced to quantify the impact of RT on the immune system [17]. This analysis suggested that higher EDIC values are associated with worse outcomes in patients with stage III non-small cell lung cancer (NSCLC). While traditional indicators such as lymphopenia, neutrophil count, and inflammatory markers such as erythrocyte sedimentation rate and C-reactive protein have been used to assess the effects of RT, these measures do not directly reflect the radiation dose absorbed by the immune system. EDIC offers a method for estimating the amount of radiation delivered to circulating immune cells, providing a more direct assessment of the impact of RT.

Given this background, we aimed to investigate the effect of radiation dose on the immune system in patients with esophageal cancer. Since the treatment fields for esophageal cancer are often large, potentially exposing a significant portion of circulating lymphocytes to radiation, the systemic effects could be considerable. Therefore, this study aims to evaluate whether EDIC can serve as an effective predictor of prognosis in patients with esophageal cancer undergoing definitive CCRT.

## Methods

### Patients

Patients diagnosed with esophageal cancer who received definitive CCRT between January 2009 and March 2022 at a single center were included. The eligibility criteria were as follows: (1) histologically confirmed squamous cell carcinoma, (2) treatment with definitive CCRT with a total dose exceeding 50 Gy, and (3) no evidence of distant metastases at diagnosis. We excluded patients who (1) underwent surgical resection before or after CCRT, (2) had a history of another malignancy, or (3) had previously received RT.

Initial staging evaluations included upper gastrointestinal endoscopy with histological biopsy; complete blood count; liver function tests; renal function tests; and computed tomography (CT) scans of the neck, chest, and abdomen; and positron emission tomography (PET)-CT scans. Endoscopic ultrasound was used to confirm the T stage in selected patients, based on the physician’s decision. This study was approved by the Institutional Review Board of our institution. The requirement for informed consent was waived due to the retrospective nature of the study. Data were collected between February 28, 2023, and May 23, 2023. Individual participants could not be identified during or after data collection.

### Treatment

For RT planning, CT simulation was performed in the supine position, with both arms positioned above the head. Simulation CT was used to delineate the gross tumor volume (GTV) with reference to the initial PET-CT for defining the primary esophageal tumor and gross nodes as the primary GTV and nodal GTV, respectively. The clinical target volume (CTV) was generated by expanding the primary GTV by 4–5 cm superiorly and inferiorly and by 1 cm radially. Nodal GTVs were expanded by 0.5–1 cm in all directions to generate a CTV. Elective nodal irradiation was considered in the supraclavicular, periesophageal, mediastinal, and perigastric areas, based on the esophageal tumor location.

The median RT dose was 5940 cGy (range: 5040–7000 cGy), with a daily fractional dose of 180 cGy, administered five times per week. The initial plan, encompassing the total target volume, delivered up to 4500 cGy, and a boost dose was delivered to the gross tumor and lymph nodes. The concurrent chemotherapy regimens included 5-FU and cisplatin (95.3%), cisplatin and carboplatin (3.7%), and weekly cisplatin (1.9%).

### Calculation of EDIC

EDIC was calculated using a model developed by Jin et al. as a function of the number of radiation fractions and mean doses to the lungs, heart, and whole body [17].


$$\text{EDIC}=\text{0}.\text{12}\times \text{MLD}+\text{0}.\text{08}\times \text{MHD}+[\text{0}.\text{45}+\text{0}.\text{35}\times \text{0}.\text{85}\times {\left(\text{number of fractions}/\text{45}\right)}^{\text{1}/\text{2}}]\times \text{ITDV}/(\text{61}.\text{8}\times {\text{10}}^{\text{3}})$$

** MLD, MHD, and ITDV refer to the mean lung dose, mean heart dose, and integral total dose volume, respectively.

**ITDV was calculated b multiplying the whole-body volume by the whole-body mean dose.

** 61.8 × 10^3^ (cm^3^) represents the average total body volume, assuming an average weight of 63 kg (140 lbs) and density of 1.02 g/cm^3^.

Dosimetric data, including mean lung dose, mean heart dose, and whole-body mean dose, were collected for EDIC calculations. The fusion of PET-CT and planning CT allowed for the acquisition of whole-body volume and whole-body mean dose by delineating the whole-body contour on PET-CT.

### Statistical analysis

Patients were stratified into two groups based on their EDIC values. The cut-off value for EDIC was determined using a maximally selected log-rank test based on progression-free survival (PFS), which identified 4.28 Gy as the optimal threshold (*p* = 0.042). Patients with an EDIC value >4.28 Gy were classified as the high EDIC group, while those with an EDIC value ≤4.28 Gy were classified as the low EDIC group.

The baseline characteristics of the two groups were compared using the t-test for continuous variables and chi-square test for categorical variables. Overall survival (OS) was defined as the time from the start of RT to death from any cause or last follow-up. PFS was defined as the time from the start of RT to disease progression at any site, death from any cause, or the last follow-up. Survival outcomes were estimated using the Kaplan-Meier method, and comparisons were made using the log-rank test. The Cox regression model was used to calculate the hazard ratios (HR) and corresponding 95% confidence intervals (CIs) for both univariate and multivariate analyses. Toxicities, including radiation pneumonitis and esophagitis, were assessed according to the Common Toxicity Criteria for Adverse Events v5.0. Statistical significance was set at *p* < 0.05. All statistical analyses were conducted using R software version 4.0.5 (R Development Core Team, Vienna, Austria).

## Results

A total of 107 patients were included in the analysis, with 65 and 42 patients in the low and high EDIC groups, respectively. Baseline patient characteristics, as shown in Table 1, were well balanced between the groups for factors such as age, sex, smoking history, clinical T stage, and prescribed RT dose. However, tumor location and clinical N stage differed significantly. The high EDIC group had a higher proportion of middle thoracic (38.1% vs. 20.0%) and lower thoracic esophageal cancers than the low EDIC group (52.4% vs. 23.1%, *p* < 0.001). Node-positive tumors were also more common in the high EDIC group than in the low EDIC group (83.3% vs. 53.8%, *p* = 0.003).

**Table 1:** Patient Baseline Characteristics According to the EDIC Group.

Characteristic - No. (%)	Total (n = 107)	Low EDIC (n = 65)	High EDIC (n = 42)	*p*-value
Age, year				0.684
≤ 65	37 (34.6%)	21 (32.3%)	16 (38.1%)	
> 65	70 (65.4%)	44 (67.7%)	26 (61.9%)	
Gender				1.000
Male	94 (87.9%)	57 (87.7%)	37 (88.1%)	
Female	13 (12.1%)	8 (12.3%)	5 (11.9%)	
Smoking history				0.385
No	21 (19.6%)	15 (23.1%)	6 (14.3%)	
Yes	86 (80.4%)	50 (76.9%)	36 (85.7%)	
Tumor location				<0.001
Cervical	15 (14%)	14 (21.5%)	1 (2.4%)	
Upper	26 (24.3%)	23 (35.4%)	3 (7.1%)	
Middle	29 (27.1%)	13 (20.0%)	16 (38.1%)	
Lower	37 (34.6%)	15 (23.1%)	22 (52.4%)	
Clinical T stage				1.00
cT1-3	14 (13.1%)	56 (86.2%)	37 (88.1%)	
cT4	93 (86.9%)	9 (13.8%)	5 (11.9%)	
Clinical N stage				0.003
cN0	37 (34.6%)	30 (46.2%)	7 (16.7%)	
cN1-3	70 (65.4%)	35 (53.8%)	35 (83.3%)	
RT dose				1.00
< 59.4Gy	15 (14%)	9 (13.8%)	6 (14.3%)	
≥ 59.4Gy	92 (86%)	56 (86.2%)	36 (85.7%)	
EDIC	3.77 ± 1.37	2.91 ± 0.91	5.1 ± 0.77	<0.001

EDIC, effective dose to the immune cells; RT, radiotherapy.

**Fig 1:**
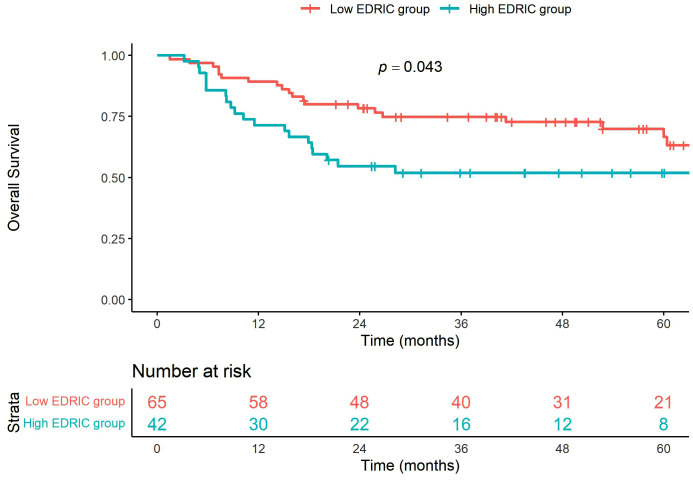
Overall survival curves according to the EDIC group.

The median follow-up time was 39 months (range: 1.5–155 months), with a median follow-up of 52.8 months for surviving patients. The 2- and 5-year OS rates for the entire cohort were 69.0% and 60.6%, respectively. The median OS was not reached. A significant difference in 5-year OS was observed between the high EDIC and low EDIC groups (51.9% vs. 66.6%, *p* = 0.043) (Fig 1). In the univariate Cox regression analysis, both old age and high EDIC group were significantly associated with poor OS (*p* = 0.017 and *p* = 0.046, respectively). Multivariate analysis showed that the high EDIC group remained an independent predictor of poor OS (HR: 2.06, 95% CI: 1.1–3.86, *p* = 0.024). Age also remained significant (HR: 2.79, 95% CI: 1.22–6.4, *p* = 0.015) (Table 2).

**Table 2:** Prognostic Factors Associated with Overall Survival.

Variable	Univariate (*p*) Hazard ratio (95% CI)	Multivariate (*p*) Hazard ratio (95% CI)
Age, year	0.017	0.015
≤ 65	1	1
> 65	2.71 (1.2-6.13)	2.79 (1.22-6.4)
Gender	0.472	
Male	1	
Female	0.68 (0.24-1.92)	
Smoking history	0.585	
No	1	
Yes	0.81 (0.39-1.71)	
Clinical T stage	0.295	
cT1-3	1	
cT4	1.88 (0.58-6.09)	
Clinical N stage	0.788	
cN0	1	
cN1-3	1.09 (0.57-2.09)	
RT dose	0.044	
< 59.4Gy	1	
≥ 59.4Gy	0.47 (0.22-0.98)	
EDIC group	0.046	0.024
Low group	1	1
High group	1.88 (1.01-3.5)	2.06 (1.1-3.86)

CI, confidence interval; EDIC, effective dose to the immune cells; RT, radiotherapy.

**Fig 2:**
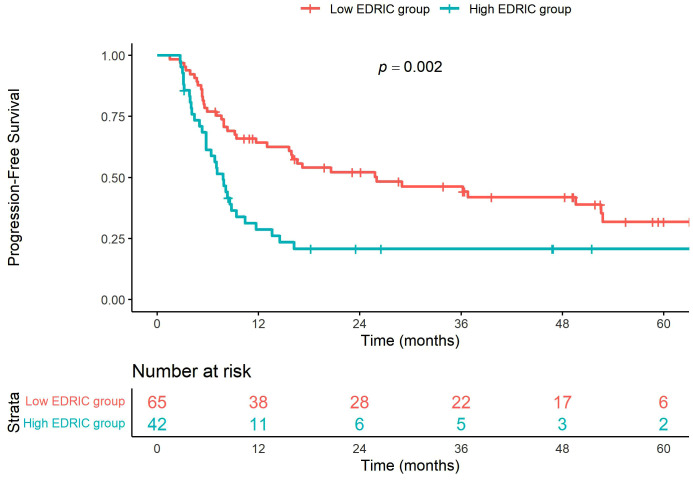
Progression-free survival curves according to the EDIC group.

Regarding PFS, the 5-year PFS rate was significantly lower in the high EDIC group than in the low EDIC group (20.8% vs. 31.8%, *p* = 0.002) (Fig 2). Univariate Cox regression analysis identified the clinical N stage and EDIC group as significant factors associated with PFS (*p* = 0.002 and *p* = 0.002, respectively). In multivariate analysis, the EDIC group showed statistical significance (HR: 1.7, 95% CI: 1.04–2.78, *p* = 0.034), with clinical N stage being a significant factor (HR: 2.0, 95% CI: 1.15–3.48, *p* = 0.014) (Table 3).

**Table 3:** Prognostic Factors Associated with Progression-Free Survival.

Variable	Univariate (*p*) Hazard ratio (95% CI)	Multivariate (*p*) Hazard ratio (95% CI)
Age, year	0.820	
≤ 65	1	
> 65	1.06 (0.64-1.75)	
Gender	0.695	
Male	1	
Female	1.15 (0.57-2.32)	
Smoking history	0.926	
No	1	
Yes	1.03 (0.56-1.88)	
Clinical T stage	0.349	
cT1-3	1	
cT4	1.45 (0.66-3.18)	
Clinical N stage	0.002	0.014
cN0	1	1
cN1-3	2.34 (1.38-3.97)	2.0 (1.15-3.48)
RT dose	0.207	
< 59.4Gy	1	
≥ 59.4Gy	0.67 (0.36-1.25)	
EDIC group	0.002	0.034
Low group	1	1
High group	2.08 (1.3-3.34)	1.7 (1.04-2.78)

CI, confidence interval; EDIC, effective dose to the immune cells; RT, radiotherapy.

**Fig 3:**
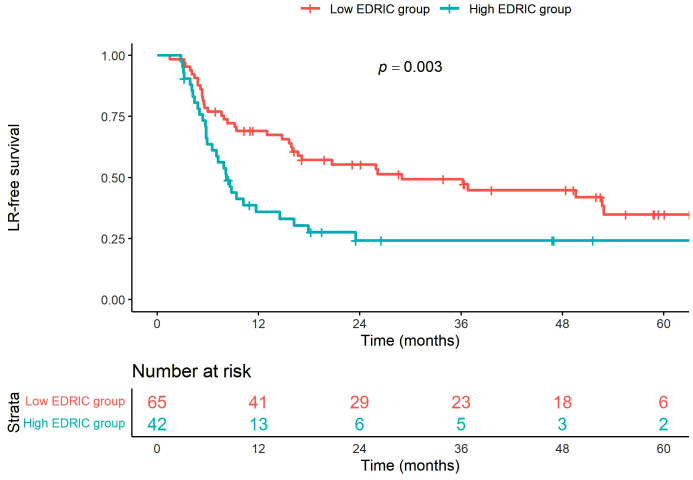
Locoregional recurrence-free survival curves according to the EDIC group.

**Fig 4:**
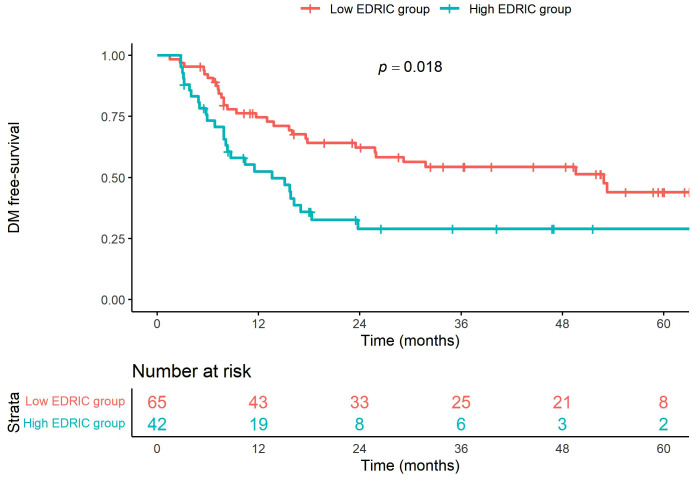
Distant metastasis-free survival curves according to the EDIC group.

Recurrence pattern analysis revealed 52 cases of locoregional failure (LRF) and 39 cases of distant metastases. The 5-year LRF-free survival rates were 24.1% and 34.9% in the high and low EDIC groups, respectively (*p* = 0.003, Fig 3). The 5-year distant metastasis-free survival rates were 29.0% and 44.0% in the high and low EDIC groups, respectively (*p* = 0.018, Fig 4).

Of the 107 patients, grade 3 or higher acute radiation esophagitis occurred in 8 patients (7.5%). Late toxicities, including esophageal stricture, fistula, and rupture, occurred in 15 patients (14.0%). Radiation pneumonitis was observed in 20 patients (18.7%).

## Discussion

In this study, we observed that elevated EDIC values were associated with poorer OS and PFS in patients with locally advanced esophageal cancer undergoing definitive CCRT. Additionally, higher rates of locoregional recurrence and distant metastasis were observed in the high EDIC group.

EDIC has been proposed as a parameter for estimating radiation exposure to circulating immune cells. It was initially introduced in secondary analyses of the RTOG 0617 trial [18], which assessed survival outcomes in patients with NSCLC treated with different radiation doses. Unexpectedly, this trial reported inferior OS in the high-dose radiation arm (74 Gy) compared to the standard-dose arm (60 Gy) [19]. Subsequent analyses suggested that increased EDIC in the high-dose group may have contributed to this outcome, potentially due to radiation-induced immune suppression [17]. EDIC was inversely correlated with OS and locoregional progression-free survival, implying that higher radiation exposure to immune cells may negatively impact clinical outcomes.

The immune system plays a pivotal role in cancer progression and treatment response. With the increasing use of immunotherapy, the importance of maintaining immune system function has become increasingly recognized [20–22]. RT can modulate immune responses in a complex manner. Although it may enhance antitumor immunity by promoting tumor cell death and the release of tumor-specific antigens, it can also exert suppressive effects. Lymphocytes, which are highly radiosensitive, can be significantly affected even by low radiation doses, with approximately 50% cell death occurring at 2 Gy [23]. Given their critical role in anticancer immunity, radiation-induced lymphopenia may negatively influence treatment outcomes [11–14].

These observations support the view that the immune system may be considered an organ at risk during radiation planning. Although leukopenia was traditionally attributed to bone marrow suppression, lymphopenia can occur even in treatment fields with minimal bone marrow involvement, likely due to radiation effects on circulating lymphocytes. This consideration is particularly relevant in thoracic malignancies, where treatment fields may have limited bone involvement but still significantly affect circulating immune cells [24,25]. Although the direct measurement of circulating lymphocytes during radiation planning is challenging, EDIC offers a useful surrogate marker [17,26].

As lymphocytes are sensitive to low radiation doses, extensive low-dose exposure across large tissue volumes can lead to lymphopenia. The heart and lungs are the major reservoirs of lymphocytes. As the central hub for circulating blood, the heart significantly influences lymphocyte exposure during irradiation [27]. Tumors located in the mid to lower esophagus can lead to considerable heart irradiation, potentially affecting circulating lymphocytes. Additionally, because all circulating blood passes through the pulmonary circulation, the lung tissue contains substantial volumes of blood and lymphocytes. Thus, broad exposure of the lungs to low-dose radiation can affect circulating lymphocytes [28]. Although the lungs are not the primary radiation targets in esophageal cancer, the longitudinal extent of the radiation field can result in substantial low-dose exposure. These factors suggest that EDIC could serve as a meaningful predictor of lymphopenia and suboptimal clinical outcomes not only in NSCLC but also in esophageal cancer [24].

The optimal EDIC cut-off value has not been definitively established and may vary depending on the cancer type. In NSCLC, radical CCRT typically involves doses of 60–66 Gy, whereas preoperative CCRT for esophageal cancer generally ranges from 43–50 Gy. Differences in prescribed radiation doses may influence EDIC values, warranting consideration of the differences in values reported in prior studies. A study investigating patients with esophageal cancer treated with CCRT at 45–50.4 Gy reported a median EDIC of 3.6 Gy [23]. In NSCLC, two studies, including RTOG 0617, reported a median EDIC of 4.7 Gy in patients treated with CCRT [17,29].

Therefore, the impact of EDIC on esophageal cancer should be investigated separately, considering its distinct role based on treatment intent and histological subtype. However, based on published studies, the cut-off values for EDIC across different studies appear to be relatively consistent. In a secondary analysis of RTOG 0617, a median value of 4.7 Gy was used as the cut-off value [17]. The study on patients with esophageal cancer indicated that an EDIC >4 Gy was associated with severe lymphopenia [23], while Freides et al. reported an increased risk of death for EDIC values between 4–6 Gy in NSCLC [29]. Our study employed a cut-off value of 4.28 Gy, which was determined using the maximally selected log-rank test, and this value was consistent with those reported in previous studies. Further investigations are necessary to determine whether the EDIC cut-off value should be adjusted for different cancer types or whether its impact on survival is independent of the cancer type itself.

Our study has a few limitations. As a single-institution retrospective study, its generalizability is limited, and the findings should be interpreted with caution. However, we attempted to minimize heterogeneity by including only patients with squamous carcinoma histology, who underwent radical CCRT and received a radiation dose of at least 50.4 Gy. In addition, Immune cell counts before, during, and after treatment were not assessed, which may have introduced unmeasured confounding. As absolute lymphocyte counts can be differentially affected by radiation, their inclusion would have allowed a more precise evaluation of the EDIC effect. Third, given the modest sample size and limited number of events, advanced adjustment strategies such as propensity score matching or inverse probability weighting were not feasible. Significant differences in tumor location and clinical N stage were observed between the two groups. Although multivariate analysis was performed, residual confounding might not be fully excluded. Larger prospective studies with immune monitoring and more rigorous adjustment methods will be required to confirm and extend our findings.

In conclusion, our study suggests that a higher EDIC is associated with poorer OS and PFS in patients with esophageal cancer undergoing definitive CCRT. The high EDIC group also showed significantly higher rates of locoregional failure and distant metastasis. Efforts are required to optimize radiation planning to lower EDIC while maintaining the prescribed radiation dose for tumor control.
